# 

**Published:** 1898-01

**Authors:** 


### INDEX TO VOLUME LII.

PAGE.

COMMUNICATIONS.

Abstract of Report and Clinics of the Southern Dental Association.............. 22
Abstract of Report of Committee on Appliances and Improvements.............. 24
An Interview.......................... 135
A Clinic on a Fusible Alloy... 168
A Method of Handling Alveolar Pyorrhea—Abstract... 478
Bridge Work. By J. L. Young, D.D.S.......................... 1
Bacteriology.......................... 401
Can Pyorrhea Alveolaris be Cured?.......................... 251
Cataphoresis vs. The Direct Application of the Galvanic Current for Obtunding Sensitive Dentine and How to Exclude Both...... 255
Does the Present Method of Teaching by Didactic Lectures Best Qualify the Student for the Practice of His Profession? By Dr. John S. Marshall...... ..... 19
Dental Faculties in Medical Schools .......................... 264
Dental Caries.......................... 368
Ethics—Professional. By H. E. Harlan, D.D.S........... 14
Electricity in the Treatment of Pulpless Teeth.......... 157
Instrument Nomenclaturo with Reference to Instrumentation ........ ......125, 174
Irregularities of the Dental Arch—Abstract............. 310
Leucoplakea—Abstract......... 451
Management of the Pulpless Teeth.......................... 469
Method of Teaching Materia Medica—Abstract.......... 507
National Dental Association, Southern Branch............ 335

PAGE.

"Orthodontia."..................... 301
Only a Baby Tooth—Abstract 412
Preliminary and Graduating Requirements—The Logical Standard.................. 465
Practical Management of Infants. By M. J. Crouch, M.D.......................... 35
Presidential Address — Abstract.......................... 316
Points .......................... 70
Personal Experiences vs. "Scientific Proof," as Factors Bearing Upon the Amalgam Question.......... 213
Preparation of Borders of Cavities for Filling......... 475
Report of the Committee on Foreign Relations of the National Association of Dental Faculties............ 551
Resolutions Adopted by the New York Institute of Stomatology................ 336
Silver Nitrate—Its Use in Dentistry. By H. Prinz, D.D.S .......................... 25
Salivary Calculi..................... 138
Some Causes of Failure in Operative Dentistry............ 171
Some Facial Deformities and Prevention—Abstract ......386
The Preliminary Education of the Dental Student. By C. G. Kuhn.................... 11
Treatment of Pulpless Teeth. By Dr. A. Schramm........ 27
The Influence and Power of Association..................... 151
The Methods of Using Gold and Tin Together...... ..... 166
Trigemenous Reflexes............ 267
The History of General Anesthesia.......................... 269
The Need of Dental Instruction in Medical Schools... 261

PAGE.

The Mouth a Human Culture Tube........................... 306
Temporo-Maxillary Articulation—Abstract............... 322
The Essential Oils and Some Other Agents—Their Antiseptic Value; Their Irritating or Non-Irritating Properties—Abstracts..... 351
The Effect of Heat on Dentine 481
The Importance of Establishing a Technic as Well as Literary Standard for College Entrance................. 501

PROCEEDINGS.

Changes in the Constitution of the Southern Dental Association, Making it a Branch of the National Dental Association......... 278
List of Committees for the Joint Meeting of the Southern Branch of the National Dental Association and the Louisiana State Dental Society........ 343
National Dental Association, Omaha, Neb., 1898... ..... 510
Cast and Model................. 511
Materia Medica and Therapeutics......................... 512
Anatomy, Pathology and Surgery ........................ 514
The Comparative Method of Teaching Dental Anatomy ............................. 522
Ohio State Dental Society...... 51
Ohio State Dental Society...... 101
Proceedings of Tri-State Dental Meeting, Held at Put-in-Bay, June 21, 22 and 23, 1898...................417–566
Resolutions Adopted by the National Dental Association at the Annual Session of 1898, and Other Miscellaneous Items................. 530
St. Augustine Meeting—Abstracts.....................279, 201

PAGE.

The Southern Branch of the National Dental Association........................... 191
The National Dental Association, Omaha, 1898.......... 483

SELECTIONS.

A Peculiar Plant.................. 74
Albert Edward, M.D............. 76
Alcohol as a Beverage........... 79
A Liberal Education............ 82
A Simple Method of Preventing the Clouding of Mirrors............................. 84
A Curious Story in Relation to Hereditary Suicide...... 92
An Attempt Likely to Fail... 137
A New Local Anesthetic........ 144
A New Element in the Blood ..............................145, 192
A Large Dussand Microphonograph ............................ 195
Alcohol as a Disinfectant..... . 196
A Wineglass of Vinegar........ 212
An Influence of the Law........ 284
Are We All Fools?.............. 334
Americans Use Medicines...... 445
Aged Parents........................ 441
After the Fracture of the Limb the Nails of the Hand on the Same Side Cease Growing................................ 506
A Whiff of Tobacco and Its Unpleasant Suggestion.... 541
Alcohol Consumption in the United States on the Decrease............................ 545
Bread and Health................ 77
Buttermilk ......................... 90
Buccelli on Effects of Tobacco 90
Be Good to Yourself.............. 91
Bone Grafting..................... 445
Causes of Bad Taste and Odor in the Mouth................... 77
Col. McKee's Bequest............ 88
Chicago Physicians Object to the Use of Cocaine......... 90
Conversational Pitfalls........... 91
Cleanliness and Health......... 444
Cement for Mending Rubber Goods............................ 443

PAGE.

Cure for Headache .......... 540
Dr. Frank Hamilton's Pithy Points .......... 78
Digestion vs. Drugs in the Treatment of Pulmonary Tuberculosis .......... 76
Don't Wet a Lead Pencil .......... 81
Diagnosis of the Diseases of Children .......... 85
Doctors and Population .......... 85
Dirty Thermometers .......... 90
Disinfection of the Mouth .......... 145
Dr. H. T. Whitney .......... 242
Dentition .......... 282
Dentistry Practice—Kind of Certificates Required in Maryland .......... 283
Divorce and Suicide .......... 496
Disinfection of Hands .......... 543
Effect of Alcoholism upon Paternity .......... 443
Enforced Higher Education .......... 538
Fruit Eating .......... 78
Farinacea in the Diet of Later Infancy .......... 439
Finishing Proximal Cement Fillings .......... 544
Formaldehyde .......... 444
Health and Athletics .......... 72
How to Succeed .......... 92
Hygiene of the Teeth .......... 543
How to Conquer Pain .......... 537
Insomnia and Suffering .......... 539
Infection Through the Air .......... 83
Improved Local Anesthesia .......... 195
Inflammation of the Sinuses of the Face .......... 321
Infantile Mortality .......... 440
Japanese Women and Suffrage 89
Japan .......... 237
Meat Eating .......... 74
Mental Fatigue and Exercise .. 89
Marriage Laws .......... 440
Music as a Sedative in Neuralgia .......... 442
Nausea from Chloroform Anesthesia .......... 92
Nerve Training .......... 285
Osteology .......... 87
Oil of Cinnamon .......... 88
Oil of Eucalyptus to Cure Chilblains .......... 237

PAGE.

On External Applications .......... 287
Orthoform–A Local Anesthetic for Open Wounds, Burns, Ulcers, etc .......... 491
Official Privileges Extended to Women Physicians .......... 495
Orthoform — A New Local Anesthetic .......... 536, 539
Preservation of Hydrogen Peroxide .......... 89
Pure Air .......... 93
Physiological Testing of Drugs 193
Ptomaines .......... 333
Pride Before a Fall .......... 490
Quick Surgical Diagnosis .......... 88
Quackery in Germany .......... 286
Recuperating Effect of Sleep ... 75
Restates the Law Relating to Malpractice .......... 281
Rumination in Man .......... 441
Substitution .......... 82
Surgeon General Sternberg .... 143
School Labor and Bodily Development .......... 285
Silver Citrate .......... 442
Shock .......... 444
The Continuity of Living Matter .......... 89
To Remove Nitrate of Silver Stains .......... 190
To Stop Nose Bleeding .......... 190
To Remove Color of Iodine and Permanganate of Potassium .......... 195
To Cure an Ingrowing Toe-nail 544
Toothache .......... 545
The Psychology of Laughing. 542
Tuberculosis of the Salivary Glands .......... 443, 196
The Camera in Astronomy .......... 309
To Mend Broken Mortars .......... 443
The Thumb and the Brain .......... 445
The Widow of the Late Sir Morell Mackenzie .......... 490
Vinegar After an Operation ... 100
What Physicians Average per Year in New York .......... 76
Worry as a Cause of Indigestion .......... 438
X Rays in Medicine and Surgery .......... 91

PAGE.

COMMENCEMENTS.

Atlanta Dental College..... ... 290
Baltimore Medical College—Dental Department........ 394
Baltimore College of Dental Surgery..................... 294
Birmingham Dental College.. 391
Chicago College of Dental Surgery............................ 288
Columbian Dental College..... 296
Cincinnati College of Dental Surgery........................ 337
Central College of Dentistry.. 395
Detroit College of Medicine... 337
Howard University — Dental Department.................. 396
Indiana Dental College......... 295
Kansas City Dental College.... 343
Meharry Medical College—Dental Department......... 297
Marion-Sims College of Medicine—Dental Department 338
Missouri Dental College........ 393
Northwestern University Dental School.................... 340
New York Dental School...... 395
National University — Dental Department ......... ........ 394
Northwestern College of Dental Surgery.................... 391
New York College of Dentistry .............................. 390
Ohio College of Dental Surgery 338
Pittsburg Dental College....... 296
Philadelphia Dental College.. 292
Pennsylvania College of Dental Surgery.................... 290
Royal College of Dental Surgeons of Ontario............ 392
Southern Medical College—Dental Department......... 390
Tennessee Medical College—Dental Department........ 287
Tacoma College of Dental Surgery............................ 392
University of Buffalo........... 288
University of Maryland—Department of Dental Surgery .. ......................... 291
University of Minnesota........ 342

PAGE.

University of Michigan—Dental Department............. 339
University of Pennsylvania—Dental Department......... 331
University of Colorado—Dental Department.............. 394
University of Denver—Dental Department.................. 396
Western Dental College ........ 294
Western Reserve University—Dental College............... 296
Washington Dental College.... 396

EDITORIAL.

A Remarkable Man............... 99
American Medical Association—Dental Section........... 240
A Beautiful Handle............. 300
Appointment Dental Board... 347
American Medical Association—Section on Stomatology 347
An Important Request.......... 349
A Very Proper Thing........... 498
A New College.................... . 550
Bibliographical ............546, 200
Close of the Year................. 592
Correction..... ....................... 200
Dentists in the Army........... 344
First Annual Meeting of the National Dental Association ......... .... ............ 299
Information ........................ 548
Matrimonial ........................ 198
Meetings ............................. 346
National Dental Association Meeting ..................349, 400
Ohio State Dental Society..98, 499
Professional Recruits........... 397
Suspension American Dental Weekly......................... 549
The Register....................... 49
The Odontographic Society of Chicago ................. ...... 147
Tri-State Meeting............348 148
The American Dental Weekly 199
The Dental News... .............. 198
The Eastern Indiana Dental Association.................... 198
The Indiana Dental Journal... 199
The Southern Branch of the National Dental Association ....... ....................... 199

PAGE.

The National Dental Association ....................... 497, 350
Division of the East .......... 200
The National Dental Technic Association .......... ........ 591
Transfer of Students ............ 242
The First Subscribers of the First Dental Journal ...... 243
The Annual Meeting of the National Association of Dental Faculties ....... 300, 400
The National Association of Dental Faculties ........ 496, 350
The Bi-State Dental Meeting 399
To the Omaha Meetings ........ 399
The Board of Dental Examiners ......... ....................... 550
Why Did He Say This? ......... 446

NOTICES.

American Dental Society in Europe ....................... 145
Illinois State Dental Society... 146
Next Meeting of the American Medical Association, June 7th to 10th, 1898 ............ 347
Planting, or Implantation; Who First Gave This Operation to the Profession? 97

PAGE.

Program of the Section on Stomatology of the American Medical Association at Denver, Colo., June 7–10, 1898 ...................... 240
Second Tri-State Dental Meeting, Hotel Victory, Put-in-Bay, Lake Erie, Ohio, June 21st, 22d, 23d, 1898.. 237
Tenth Anniversary of the Odontographic Society of Chicago, Ills .................. 97
The Eleventh Annual Session of the Washington State Dental Society ................ 298
The National Association of Dental Examiners ........ 445
To the Members of the Ohio State Dental Society ....... 545
University of Tennessee, Dental Department, Nashville, Tenn., Alumni Clinic 50

OBITUARY.

Dr. H. P. Southwick ............. 448
Died Lately in Holland ........ 499
In Memory of Dr. W. B. Chambers ............. ....... 150
Mr. Ernest Hart .................. 149

### NOTICES.

### University of Tennessee, Dental Department, Nashville, Tenn., Alumni Clinic.

*Dear Doctor:*—You are invited to attend the Alumni Clinic which will be held in the College Building of the Dental Department of the University of Tennessee, at Nashville, January 26 and 27, 1898. The faculty respectfully request that you come prepared to contribute a clinic, paper, or models representing some dental instrument or operation. If not prepared to make either of the above-mentioned contributions to the interest of the meeting, we should be glad if you would honor us with your presence. We are expecting a number of distinguished operators from other cities besides the clinicians from the alumni society, and we feel sure that the occasion will be one of much interest and profit. Drs. Gordon White and J. M. Glenn will superintend the clinics. Will you please inform us at once of what nature your contribution will be?

We beg to remain very truly and cordially yours,

UNIVERSITY OF TENNESSEE, DENTAL DEPARTMENT.

*December* 15, 1897.

### CONTENTS.

COMMUNICATIONS.

EDITORIAL

The "Register". ...... 49

NOTICES.

University of Tennessee, Dental Department, Nashville, Tenn., Alumni Clinic ...... 50

---

### THE DENTAL REGISTER,

A MONTHLY MAGAZINE DEVOTED TO THE INTERESTS OF THE

### DENTAL PROFESSION.

*Terms Reduced to* $2.00 *Per Year. Single Copies,* 25 *Cents.*

**EDITED BY PROF. J. TAFT, D.D.S.**

—AND—

**Published by SAM'L A. CROCKER & CO., Ohio Dental and Surgical Depot.**

The volume commences in January and closes in December.

The REGISTER being issued monthly is always fresh, therefore Indispensable to the practitioner who desires to keep posted up with the new inventions and improvements which are daily developed. Neither expense nor effort will be spared to give value and variety to its contents. Articles will be illustrated with well executed engravings whenever they will add to the interest or assist to explanation.

Its extensive circulation and frequency of issue make it one of the BEST DENTAL ADVERTISING MEDIUMS in the United States.

All subscriptions must begin with January or July.

Local or special notices, five lines or less, will be inserted for $3 each insertion; over five lines, 50 cts. per line.

All advertising bills payable in advance, or at furthest, after the issue of the first number containing the advertisement. All transient advertisements to be paid for in advance.

☞CONTRIBUTIONS SOLICITED.

Exclusively Editorial Communications should be addressed to the Editor.

The latest number of the REGISTER may be ordered with or without other goods at any time, and all letters should be addre[illegible]

